# CHANGES IN GENERAL SELF-EFFICACY FOLLOWING THE WITS WELLNESS PROGRAM: PRELIMINARY FINDINGS FROM THE 12-WEEK RCT

**DOI:** 10.1093/geroni/igac059.2724

**Published:** 2022-12-20

**Authors:** Neha Gothe, Molly Hofer, Chelsey Byers, Laura Payne, Julie Bobitt

**Affiliations:** University of Illinois at Urbana Champaign, Urbana, Illinois, United States; University of Illinois Extension, Chicago, Illinois, United States; University of Illinois Extension, Champaign, Illinois, United States; University of Illinois at Urbana Champaign, Urbana, Illinois, United States; University of Illinois at Chicago, Chicago, Illinois, United States

## Abstract

Self-efficacy among older adults has been associated with better sleep, increased vitality, decreased pain and discomfort as well as overall satisfaction with life. Interventions designed to increase general self-efficacy can therefore have wide-ranging effects on individual’s health as well as reduce the burden on healthcare systems. In this abstract we present preliminary data examining the impact of Wits Wellness on general self-efficacy among participants. Middle aged and older adults, Nf285 (Mean age 65.58 yrs, males=28) were randomized to either a Wits Wellness group or a Waitlist Control for 12-weeks. The 12-week wellness program addresses multiple factors including physical activity, stress, sleep, social isolation and diet with the goal of empowering older adults to make healthier lifestyle choices. At baseline and end of the 12-week trial, the 10-item PROMIS general self-efficacy scale was used to assess global self-efficacy which reflects a problem-solving approach despite perceived obstacles and challenges. Older participants aged 65 and above, who attended 8 or more weeks (Nf123, n=63 in Wits Wellness group) of the program demonstrated significant increases in their general self-efficacy. Results showed a significant group*time effect favoring the Wits Wellness intervention (F(1,120)=4.10, p=.045, partial eta2=.03). The intervention was most effective in boosting confidence among the older adults within the trial (>65 yrs.). These findings demonstrate preliminary efficacy of the Wits Wellness program and are significant as general self-efficacy is considered an important moderator of healthy adaptation to illness which is critical in old age. Funded by the Midwest Roybal Center grant P30AG022849.

